# Effect of Molecular Weight and Protein Content on the Air–Water Interfacial and Foaming Properties of Soybean Soluble Polysaccharides

**DOI:** 10.3390/foods15081272

**Published:** 2026-04-08

**Authors:** Yujian Li, Guijiang Liang, Zhaojun Wang, Maomao Zeng, Zhiyong He, Qiuming Chen, Fang Qin, Jie Chen

**Affiliations:** 1 State Key Laboratory of Food Science and Resources, Jiangnan University, Wuxi 214122, China; lyjdgzyx@163.com (Y.L.); liangguijiang0103@163.com (G.L.); zhaojun.wang@jiangnan.edu.cn (Z.W.); mmzeng@jiangnan.edu.cn (M.Z.); zyhe@jiangnan.edu.cn (Z.H.); chenqm@jiangnan.edu.cn (Q.C.); qfflast@sina.com (F.Q.); 2 School of Food Science and Technology, Jiangnan University, Wuxi 214122, China

**Keywords:** soybean soluble polysaccharides, protein, interfacial rheology, foaming properties

## Abstract

This study systematically investigated the influence of molecular weight (MW) and protein content (PC) on the interfacial behavior and foaming properties of soluble soybean polysaccharide (SSPS), aiming to elucidate the structure–function relationship for the targeted design of SSPS-based foam stabilizers. The results demonstrated that the low-MW group, particularly the LH sample (low MW, high PC), exhibited the highest foam expansion (FE = 272.5%), attributed to its smallest particle size, lowest zeta potential, and minimal surface tension, which facilitated rapid adsorption at the interface. Interfacial rheology revealed that all SSPS samples formed an elastic-dominated interfacial film (G′ > G″). The HM sample (high MW, moderate PC) showed the most rapid increase in G′ and the highest mechanical strength, while the LH sample (low MW, high PC) exhibited the strongest elastic response within the low-MW group, which contributed to its relatively high foam stability (FS = 69.9%). The interfacial viscoelasticity and foaming performance of SSPS are synergistically governed by its MW and PC. Low MW facilitates rapid adsorption and superior foam expansion, while high PC enhances interfacial film elasticity. Moreover, the long-term stability of foam depends not only on reduced interfacial tension but more critically on the mechanical strength and viscoelasticity of the interfacial film. These findings provide a crucial theoretical basis for optimizing SSPS applications in aerated foods.

## 1. Introduction

As an interface-dominated multiphase dispersion system, foam effectively imparts unique textural, sensory, and visual qualities to aerated foods, such as whipped cream, ice cream, cakes [1], and beer, due to its distinct air–liquid interfacial structure, thereby attracting considerable interest in the food industry. Foam, as a thermodynamically unstable system, owes its instability to the high specific surface area and surface free energy. To reduce the system’s energy, amphiphilic molecules—such as surfactants, solid particles, or natural proteins—adsorb at the air–water interface [2]. By modulating the properties of the interfacial film, kinetic stability can be achieved. Natural proteins, leveraging their amphiphilic structure, rapidly adsorb at the interface to form a viscoelastic film, thereby enhancing foam stability [3]. This process addresses the thermodynamic instability of foams over the shelf life of food products. Plant proteins act as effective stabilizers for food foams due to their amphiphilic nature, which promotes adsorption at the air–water interface and the formation of a strong, viscoelastic film [4]. Because they are widely available, environmentally friendly, and nutritious, these proteins have received significant attention for developing sustainable food systems. Nevertheless, most native proteins exhibit limitations in conferring long-term stability to foam systems, with limited foamability and inadequate stability, which restrict their practical applications [3,5]. The formation of complexes between proteins and food-grade polysaccharides has been demonstrated as an effective strategy to significantly enhance their foaming properties [6,7,8]. This approach primarily relies on the steric hindrance effects and electrostatic repulsion provided by polysaccharide chains, which collectively strengthen the mechanical strength of the interfacial film, thereby effectively retarding bubble coalescence and delaying liquid drainage processes [2].

Recent studies have demonstrated that protein–polysaccharide complexes formed via non-covalent interactions exhibit excellent adsorption and film-forming capabilities at the air–water interface, conferring significantly enhanced stability and persistence to the foam system [9,10]. SSPS is a natural anionic polysaccharide characterized by a backbone of galacturonic acid and homogalacturonic acid, with neutral side chains composed of arabinose and galactose. These highly branched side chains promote a star-like or comb-like extended conformation in aqueous solution [11,12,13]. Additionally, SSPS incorporates protein moieties bound by both covalent and non-covalent interactions [14,15]. Our previous work revealed that, under acidic conditions, high-molecular-weight (MW) SSPS with a low degree of esterification (DE) provides excellent protein-stabilizing capacity, while high-MW SSPS with moderate protein content (PC) demonstrates superior emulsifying performance [14,16], highlighting their potential as effective macromolecular stabilizers in complex food systems, such as acidified dairy beverages. It should be noted that this previous study primarily evaluated the interfacial functionality of SSPS in oil–water systems, focusing on emulsifying performance and oil–water interfacial activity. In contrast, low-molecular-weight fractions appear to play distinct roles in interfacial functionality, suggesting that the molecular characteristics of SSPS critically influence their interfacial behavior and functional properties. However, systematic investigations into the air–water interfacial behavior, interfacial rheology, and foaming mechanisms of SSPS with different MW and PC remain limited, which is the focus of the present study.

To address this gap, we hypothesize that the molecular characteristics of SSPS, particularly MW, are critical determinants of their interfacial behavior and functional properties. In particular, the independent and synergistic effects of MW and PC on interfacial adsorption kinetics, interfacial rheology, and foam stability have not yet been clearly elucidated. A deeper understanding of these structure–function relationships is essential for rationally tailoring SSPS performance in aerated systems. Therefore, this study prepares SSPS fractions with well-defined MW and PC and comprehensively characterizes their compositional features, including MW distribution, PC, particle size, ζ-potential, amino acid composition, and surface tension, together with their air–water interfacial properties and foaming performance. By correlating molecular characteristics with interfacial behavior and macroscopic foam properties, we aim to uncover the mechanistic roles of MW and PC in governing SSPS functionality. The findings are expected to provide both fundamental insights and practical guidance for designing SSPS-based stabilizers in aerated food formulations.

## 2. Materials and Methods

### 2.1. Materials

Two principal materials were employed: soybean okara and soybean meal, with compositions detailed as follows (crude protein; total dietary fiber): okara (18.7%; 73.8%) and meal (44.5%; 37.7%). These were sourced from Pingdingshan Jinjing Biotechnology Co., Ltd. in Henan, China. All additional chemicals and reagents were analytical grade.

### 2.2. Preparation of SSPS Samples

SSPS samples with different MW and PC were prepared following the protocol outlined in Table 1. The table, summarizing the compositional characteristics of the SSPS fractions, is reproduced from our previous publication [14] with minor modifica-tions where appropriate. Briefly, mixtures of soybean meal and okara at designated mass ratios were dispersed in distilled water (4%, *w*/*w*). The resulting suspension was adjusted to pH 4.0 and subjected to extraction in a high-pressure reactor at 110–130 °C for 2.5 h. The supernatant isolated following centrifugation (5400× *g*, 10 min) was first concentrated via rotary evaporation and subsequently subjected to precipitation by adding three volumes of 95% ethanol. The resultant precipitate was harvested through repeated centrifugation under identical conditions, thoroughly washed with 95% ethanol in three successive cycles, and finally dehydrated at 60 °C for 24 h.

### 2.3. Molecular Weight Determination of SSPS

For MW determination, the SSPS samples were subjected to gel permeation chromatography using a TSK-gel G5000PWXL column (Tosoh Bioscience, Tokyo, Japan). Separation was carried out on an Alliance e2695 HPLC system (Waters, Milford, USA), and the eluting components were monitored by an in-line 2414 refractive index detector [14]. The MW of the SSPS samples was determined by gel permeation chromatography relative to dextran standards. Filtered (0.45 µm) sample solutions (10 mg/mL) were analyzed using a 50 µL injection. The separation employed a 0.1 M phosphate buffer (pH 6.8) mobile phase flowing at 0.6 mL/min with the column temperature set at 40 °C. The calibration curve was constructed using dextran standards with MWs of 5.25, 13.05, 64.65, 135.35, 300.60, and 2000.00 kDa. The relationship between retention time (t) and the logarithm of MW (log Mw) was determined by linear regression, where R^2^ denotes the coefficient of determination indicating the goodness of fit. The resulting equation was: log Mw = −0.2695t + 9.1935 (R^2^ = 0.9969).

### 2.4. Protein Content Determination of SSPS

The PC in the SSPS samples was measured using the Kjeldahl method [15]. Digestion was performed in a digestion furnace (Huaye, Shanghai, China) by treating the sample with 0.4 g cupric sulfate, 6 g potassium sulfate, and 20 mL sulfuric acid. The digested sample was subsequently transferred to an automatic Kjeldahl nitrogen analyzer (Hanon, Jinan, China). The necessary reagents—including 40% sodium hydroxide, 0.01400 mol/L hydrochloric acid, and 2% (*w*/*v*) boric acid containing a mixed indicator—were loaded into the system to enable fully automated addition, distillation, titration, and data recording.

(1) $$PC\left(\%\right)=\frac{(V-V_{0})\times C\times 0.014\times F}{m}\times 100\%$$

V: Volume of standard acid consumed in titrating the digested sample, in milliliters (mL).
V_0_: Volume of standard acid consumed in titrating the blank control digested solution, in milliliters (mL).
C: Molar concentration of the standard acid, in moles per liter (mol/L).
0.014: Millimolar mass of nitrogen, i.e., the mass of 1 millimole of nitrogen is 0.014 g (g/mmol).
F: Conversion factor for nitrogen to protein (F = 6.25)
m: Mass of the sample, in grams (g).

### 2.5. Particle Size and ζ-Potential Measurements

The particle size and ζ-potential were determined according to the method described by Ma et al. [17] and Li et al. [14]. The particle size and zeta potential of SSPS were determined using a Nano-ZS Malvern laser particle size analyzer (Malvern Instrument, Malvern, UK). SSPS solutions were diluted to 0.5% with citrate-phosphate buffer (pH 4) and filtered through a 0.45 μm membrane. Measurements were performed in triplicate at 25 °C, and the data were subsequently analyzed using the Zetasizer software(version 7.13).

### 2.6. Amino Acid Analysis

The sample digestion was carried out in a thick-walled pressure vessel under an inert atmosphere at 110 °C for 24 h using 6 M HCl containing 0.1% phenol [18]. After hydrolysis, the mixture was transferred to a volumetric flask, filtered through filter paper, and subsequently passed through a 0.22 μm membrane. The resulting hydrolysate was subjected to pre-column derivatization and analyzed on an Agilent 1100 HPLC system (Santa Clara, CA, USA) equipped with a variable wavelength detector (G1314A) and an Agilent Hypersil ODS column (5 μm, 4 mm × 250 mm, Thermo Fisher Scientific Inc, Waltham, USA) maintained at 40 °C. Proline was detected at 338 nm and 262 nm. Amino acid standards were employed to construct calibration curves for quantification. Amino acid concentrations in the samples were determined directly from these curves. Chromatographic separation was performed under the following conditions: Eluent A:27.6 mM sodium acetate (pH 7.2):triethylamine: tetrahydrofuran (500:0.1:2.5, *v*/*v*/*v*). Eluent B:80.9 mM sodium acetate (pH 7.2):methanol:acetonitrile (1:2:2, *v*/*v*/*v*). The gradient program was: 0 min, 8% B; 17 min, 50% B; 20.1 min, 100% B; 24 min, 0% B. Flow rate: 1.0 mL/min. Column temperature: 40 °C. Detection wavelength: 338 nm. Injection volume: 10 µL. This provides full details of the chromatographic conditions for reproducibility.

### 2.7. Surface Tension

The surface tension of the SSPS solutions was determined following the procedure established by Li et al. [19]. Measurements were performed on a DCAT21 automated tensiometer (Dataphysics, Berlin, Germany) employing the Wilhelmy plate method at a constant temperature of 20 °C, with sample concentrations fixed at 1% (*w*/*w*). To ensure measurement precision, the platinum plate was meticulously rinsed with deionized water and flame-cleaned at high temperature before each measurement to eliminate any organic contaminants. The instrument, with an operational range of 1–1000 mN/m, maintained an accuracy of ±0.01 mN/m throughout the experimental series.

### 2.8. Interfacial Shear Rheological Properties

The interfacial rheological properties of the SSPS solutions were assessed using a Haake Mars III rheometer (Thermo Fisher, Karlsruhe, Germany), adopting the testing approaches outlined by Li et al. [19] and Wang et al. [20]. Interfacial rheological properties were assessed through a methodology involving a Du Noüy ring constructed from platinum-iridium, with precise geometrical and inertial parameters: radius of 9.725 mm, wire diameter of 0.379 mm, and a system moment of inertia of 2 × 10^−7^ kg·m^2^. The sample consisted of a 1% SSPS solution dispensed into a borosilicate glass cell measuring 60 mm in diameter. Automated alignment positioned the ring at the air–liquid interface, maintaining an immersion depth of 0.5 ± 0.1 mm for the lower edge. Dynamic oscillatory shear measurements were executed via RheoWin software (version 4.0), whereby strain amplitudes were systematically varied from 0.01% to 100% at a fixed angular frequency (ω = 1 rad/s), facilitating continuous monitoring of the time-dependent evolution of the interfacial storage modulus (G′) and loss modulus (G″).

### 2.9. Foaming Properties

The foam expansion (FE) and foam stability (FS) of SSPS were determined according to the method described by Wan [21] with modifications. Briefly, 10 mL of a 1% (*m*/*v*) SSPS solution was transferred into a 100 mL plastic measuring cylinder and whipped at 17,500 rpm for 2 min using a homogenizer (T18 digital ULTRA-TURRAX, IKA, Staufen im Breisgau, Germany). The foam expansion and foam stability were calculated using Equations (1) and (2), respectively, as follows:

(2) $$FE\left(\%\right)=\frac{\left(V_{1}-V_{0}\right)}{V_{0}}\times 100\%$$

(3) $$FS\left(\%\right)=\frac{V_{2}}{V_{1}}\times 100\%$$

The initial volume (mL) of the SSPS solution is denoted as V_0_, and V_1_ and V_2_ are the foam volumes measured immediately after homogenization and after 30 min, respectively.

### 2.10. Statistical Analysis

All experiments included at least three independent replicates, except for the amino acid analysis, which was performed only once. Data are reported as mean ± standard deviation. Using SPSS Statistics (v.25.0; IBM, Armonk, NY, USA), significant differences (*p* < 0.05) among means were assessed by one-way ANOVA followed by LSD post hoc tests.

## 3. Results and Discussion

### 3.1. Molecular Weight Distribution of SSPS

The MW distribution of the nine SSPS samples is presented in Table 2. The MW of the samples was primarily regulated by the acid extraction temperature, whereas the PC was modulated by increasing the proportion of soybean meal in the raw materials. To delineate the compositional differences among the nine SSPS samples, the percentage distribution of components across specific MW ranges (>400 kDa, 100–400 kDa, 10–100 kDa, and <10 kDa) was calculated based on the MW distribution profiles, as summarized in Table 2. Clear differences in MW composition were observed among the three MW groups (H, M, and L). Samples in the high-MW group (HH, HM, HL) exhibited a predominant proportion of macromolecular fractions (>400 kDa and 100–400 kDa), accounting for over 45% of the total components, indicating the presence of more highly polymerized polysaccharide–protein complexes. In contrast, the low-MW group (LH, LM, LL) showed a substantial reduction in these high-MW fractions and a marked increase in the 10–100 kDa fraction, suggesting enhanced molecular degradation and the formation of smaller, more soluble fragments during extraction. Within each MW group, increasing PC further altered the MW distribution. Specifically, samples with higher PC consistently exhibited a greater proportion of the 10–100 kDa fraction compared with their counterparts with similar overall MW. This phenomenon may be attributed to the relatively lower MW of protein components compared to polysaccharide chains, thereby shifting the overall distribution toward intermediate MW regions. The increased protein incorporation likely disrupted large polysaccharide aggregates and promoted the formation of smaller protein–polysaccharide conjugates.

### 3.2. Particle Size and ζ-Potential Analysis

**Figure 1:**
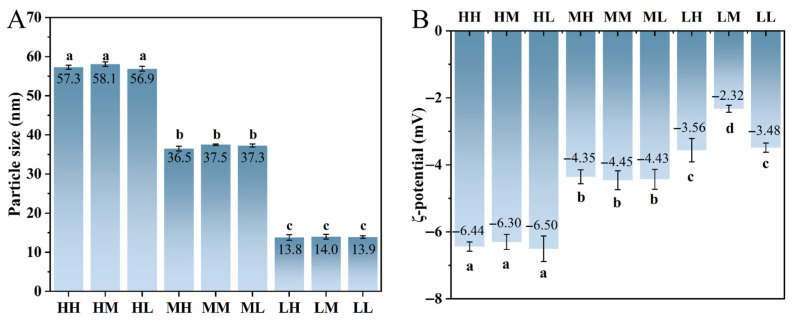
Particle size (**A**) and ζ-potential (**B**) of SSPS samples. Different letters indicate significant differences (*p* < 0.05).

Particle size and ζ-potential were jointly analyzed to elucidate the colloidal characteristics and dispersion stability of the SSPS samples. As shown in Figure 1A, the average particle size decreased progressively with decreasing MW, from approximately 57–58 nm in the high-MW group to 36–38 nm in the medium-MW group and further to 13–14 nm in the low-MW group. This gradual reduction in particle size suggests that lower-MW SSPS formed smaller aggregates and exhibited improved dispersion, likely due to reduced intermolecular entanglement and steric association. Notably, particle size correlated well with the proportion of high-MW fractions, confirming that larger macromolecular assemblies tended to associate into bigger colloidal particles. The ζ-potential results at pH 4.0 (Figure 1B) further clarified the stability differences among samples. For samples within the same MW group, variations in PC had only a minor influence on the ζ-potential, indicating that molecular size rather than protein proportion predominantly governed surface charge characteristics. In contrast, significant differences were observed among MW groups. The high-MW samples exhibited significantly higher absolute ζ-potential values (approximately −6.5 mV) compared with the medium- and low-MW groups (*p* < 0.05), indicating stronger electrostatic repulsion between particles. Combining the particle size and ζ-potential results, it can be inferred that the enhanced surface charge of high-MW SSPS provided stronger electrostatic stabilization, which helped suppress particle aggregation and maintain relatively uniform dispersions despite their larger molecular size. Meanwhile, the smaller particle sizes observed in the low-MW samples were primarily attributed to their reduced molecular dimensions rather than electrostatic effects. Therefore, the colloidal properties of SSPS were governed by the combined contributions of molecular size and interparticle electrostatic interactions. Collectively, these findings demonstrate that extraction conditions and protein incorporation effectively regulate the molecular architecture, surface charge, and aggregation behavior of SSPS, thereby modulating their colloidal stability. Such structural and physicochemical differences are expected to significantly influence their interfacial adsorption behavior and functional performance in food systems, particularly in foam and emulsion stabilization [22].

### 3.3. Amino Acid Composition Analysis

The amphipathic nature of proteins, determined by the relative proportion of hydrophobic and hydrophilic residues in their sequences, governs their surface hydrophobicity and electrostatic properties. These characteristics collectively facilitate protein adsorption at the air–water interface and contribute to interfacial film formation, which is essential for foam formation and stability [23,24]. Hydrophobic amino acids mainly include alanine, valine, leucine, isoleucine, proline, phenylalanine, methionine, and tryptophan. As summarized in Table 3, samples with higher PC generally exhibited a greater proportion of hydrophobic amino acids, whereas MW showed no significant influence on the overall hydrophobic amino acid content. Specifically, the hydrophobic amino acid contents of the HH, MH, and LH samples were 28.29%, 30.22%, and 28.94%, respectively. A higher level of exposed hydrophobic residues is theoretically favorable for foaming performance, as these groups reduce interfacial free energy and promote rapid adsorption and rearrangement at the interface [22]. Although MW had little effect on total hydrophobic amino acid content, variations in amino acid composition were observed among samples with similar PC but different MW. These changes in amino acid composition may be associated with the acid extraction and hydrolysis conditions applied during SSPS preparation, which could preferentially modify or degrade specific protein fractions. Such compositional differences may further influence the charge characteristics, interfacial adsorption behavior, and ultimately the foaming functionality of SSPS samples.

### 3.4. Surface Tension Analysis

**Figure 2:**
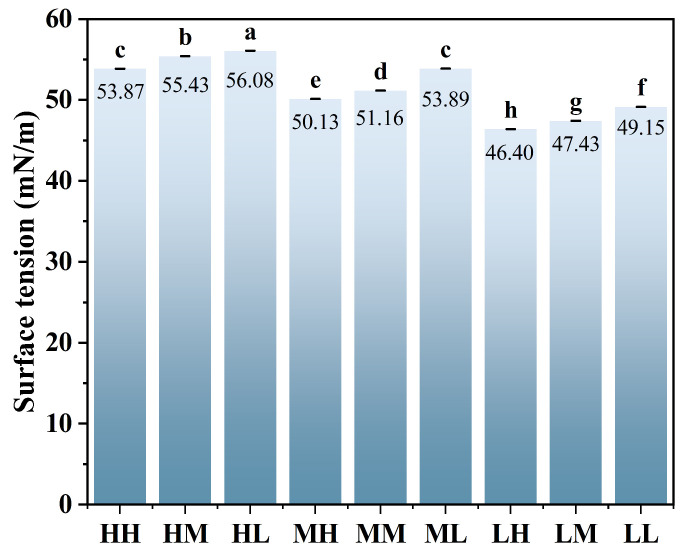
Surface tension measurements for 1% (*w*/*v*) SSPS solutions at 20 °C. Different letters indicate significant differences (*p* < 0.05).

Figure 2 presents a comparison of the surface tension values for the nine SSPS samples, each measured at a concentration of 1%. Low-molecular-weight SSPS exhibited a pronounced reduction in surface tension. Specifically, the 1% (*w*/*v*) solutions of LH, LM, and LL showed surface tension values of 46.40, 47.43, and 49.15 mN/m, respectively, which were lower than those of the medium- and high-MW groups (MH: 50.13, MM: 51.16, ML: 53.89 mN/m; HH: 53.87, HM: 55.43, HL: 56.08 mN/m). Among these, the LH sample had the lowest surface tension, whereas HM exhibited the highest among all samples. During foam formation, the expansion of the liquid surface area leads to an increase in the system’s surface free energy [25], whereas foam collapse results in a reduction in this energy. When the surface tension of the liquid is low, the increase in surface free energy during foaming is relatively small, and the energy released upon collapse is also limited. Although reduced surface tension facilitates foam formation, it alone cannot fully account for foam stability. Low interfacial tension provides only a preliminary condition for foam stabilization; long-term foam stability ultimately depends on the mechanical strength and viscoelasticity of the air–liquid interfacial film [3]. The significantly lower surface tension of the LH sample likely facilitated its rapid adsorption at the air–water interface, promoting foam formation and resulting in high foam expansion. Conversely, despite its relatively high surface tension, the HM sample may form a stronger interfacial film due to its higher MW, which could contribute to its relatively better foam stability over time. These observations indicate that while reduced surface tension favors initial foam formation, sustained foam stability depends on the mechanical properties of the interfacial film, which are influenced by both MW and PC.

### 3.5. Air–Water Interfacial Rheological Behavior Analysis

**Figure 3:**
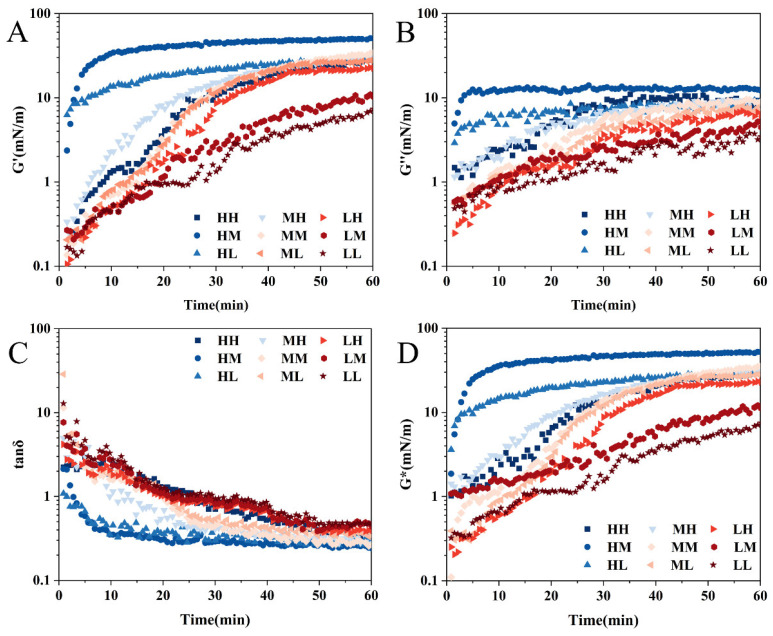
Temporal evolution of the rheological parameters for SSPS samples at the air–water interface: (**A**) storage modulus (G′), (**B**) loss modulus (G″), (**C**) loss tangent (tan δ), and (**D**) complex modulus (G*).

The foaming performance of proteins is fundamentally governed by their adsorption, diffusion, and rearrangement behaviors at the interface between the aqueous and gaseous phases [5]. An in-depth analysis of the interfacial rheological properties of protein–polysaccharide complexes at this interface is of critical importance for elucidating the stabilization mechanisms of foam systems [26]. As illustrated in Figure 3, the storage modulus (G′) of all SSPS samples consistently exceeded the loss modulus (G″) from the initial adsorption stage, indicating the formation of an interfacial film with predominantly elastic characteristics. Notably, the high MW of SSPS with moderate PC (sample HM) exhibited the most rapid increase in G′, suggesting the development of a cohesive and mechanically robust interfacial layer. In contrast, the low MW SSPS with high PC (sample LH) demonstrated the strongest interfacial structural strength among the three low MW samples, highlighting the significant role of PC in modulating the viscoelastic properties of low MW fractions. The viscoelastic characteristics of the interfacial film can be characterized by the loss factor (tan δ), which is defined as the ratio of the viscous modulus to the elastic modulus. This ratio intuitively indicates whether the interfacial film is predominantly viscous-dominated or elastic-dominated [27]. Generally, a lower tan δ value signifies a stronger elastic response of the interfacial film. During the adsorption process, the tan δ values for the HM and HL SSPS samples were below 1 from the initial stage, indicating that the G″ was consistently lower than the G′, and the interfacial film was primarily elastic in nature. Over time, all samples exhibited tan δ < 1 after 30 min, indicating an elastic-dominated state. Within the low MW group, the LH sample showed the lowest tan δ value, suggesting its interfacial film possessed a relatively stronger elastic response. Furthermore, the combination of the complex modulus (G*) and G′ trends revealed a high degree of consistency, demonstrating that the HM sample formed an interfacial film with the highest mechanical strength. Among the low MW samples, the LH sample also exhibited a relatively stronger interfacial film structure. Collectively, these findings demonstrate that the interfacial viscoelastic behavior of SSPS is not only governed by MW and PC but is also synergistically modulated by the interaction patterns between the protein and polysaccharide chains.

**Figure 4:**
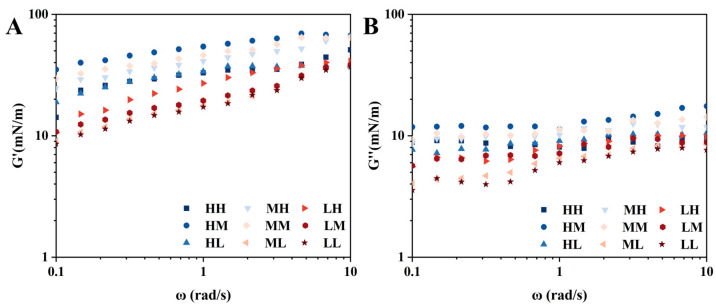
Frequency sweep measurements of SSPS samples at the air–water interface. (**A**): G’; (**B**): G”.

The frequency sweep results presented in Figure 4 demonstrate that within the angular frequency range of 0.1 to 10 rad/s, both the G′ and the G″ gradually increased with rising frequency. Furthermore, G′ consistently remained higher than G″ throughout the tested range, indicating that the interfacial film exhibited elastic-dominated rheological behavior across the entire frequency domain investigated.

**Figure 5:**
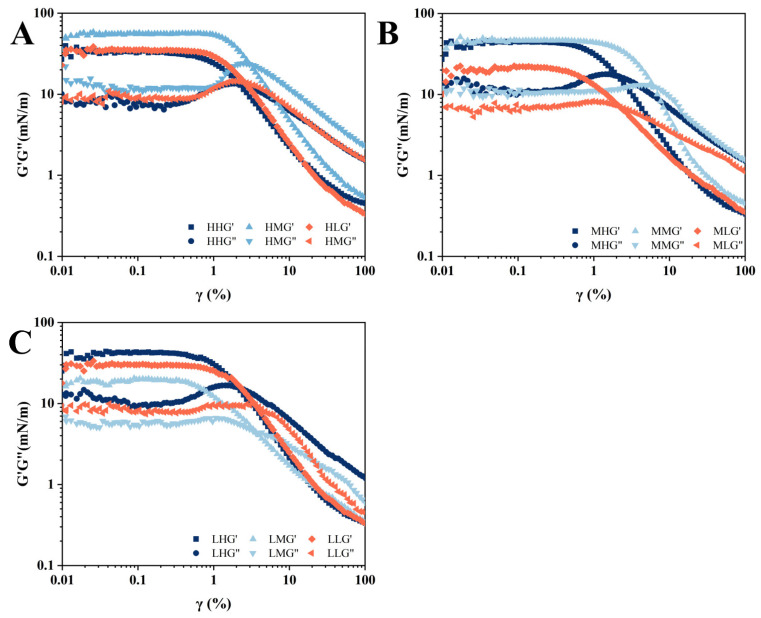
The strain-dependent behavior of the interfacial G′ and G″ is presented for SSPS samples at the air–water interface. (**A**): HH, HM, HL; (**B**): MH, MM, ML; (**C**): LH, LM, LL.

Figure 5 presents the amplitude sweep results of the SSPS samples over a strain range of 0.1% to 100%, which were conducted to investigate the fracture behavior of the interfacial film. Among the nine samples, the HM sample exhibited the highest G′ within the linear viscoelastic region, indicating the formation of the most structurally stable air–water interfacial film. As the strain increased beyond the linear viscoelastic regime, both G′ and the G″ of all samples began to decrease until a crossover point was observed. Beyond this crossover point, G′ decreased below G″, indicating that the interfacial film microstructure was disrupted under high shear strain, resulting in the loss of elastic dominance and a transition toward predominantly viscous, liquid-like behavior [28]. Within the three low MW SSPS samples, the LH sample displayed the highest G′ value; however, its interfacial network integrity was lost at approximately 2.5% strain, reflecting a structural strength considerably lower than that of the high MW samples.

### 3.6. Foaming Properties Analysis

**Figure 6:**
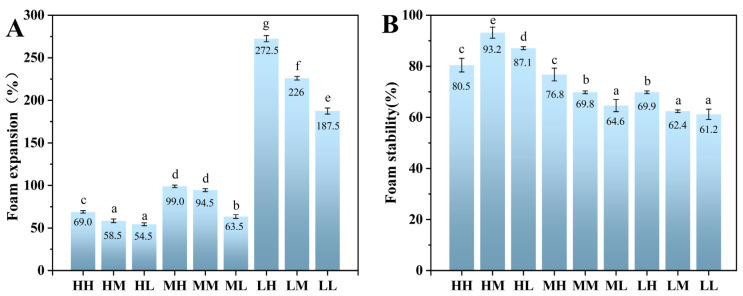
Foam expansion (**A**) and foam stability (**B**) of SSPS samples at 1% (*m*/*v*). Different letters indicate significant differences (*p* < 0.05).

The foamability and stability of foams are key determinants of the quality and sensory properties of aerated foods. A comprehensive evaluation of foam performance requires the integrated analysis of multiple parameters, including foam volume, drainage rate, bubble size distribution, and interfacial film strength, which collectively reflect the interactions among destabilizing factors and their macroscopic manifestations [5]. As shown in Figure 6, the low-MW group exhibited the most favorable foaming properties, with the LH sample demonstrating the highest performance. Specifically, the LH sample achieved foam expansion (FE) and foam stability (FS) values of 272.5% and 69.9%, respectively. This superior performance can be attributed to a combination of factors. The LH sample had the smallest particle size, the lowest ζ-potential, and the minimal surface tension within the low-MW group. These characteristics collectively facilitated rapid adsorption at the air–water interface, promoting the formation of a cohesive interfacial film. Moreover, the high PC of the LH sample likely contributed to the formation of a viscoelastic interfacial layer, which enhanced the mechanical strength of the foam film and reduced the rate of bubble coalescence and drainage. The small particle size enabled faster diffusion to the interface, while the moderate surface charge reduced excessive repulsion between particles, ensuring close packing and stabilization of the interface. Together, these factors resulted in a foam with both high expansion and long-term stability. Interestingly, while the LH sample exhibited the highest foam expansion, the HM sample (high MW, moderate PC) demonstrated the best overall foam stability (FS), as indicated by its superior resistance to bubble coalescence and slower drainage over time. This enhanced stability is likely due to the formation of a stronger and more elastic interfacial film, as confirmed by interfacial rheology measurements showing the fastest increase in G′ and the widest linear viscoelastic region in HM. Overall, these findings indicate that both MW and PC are critical determinants of SSPS foaming performance. Low MW promotes better dispersibility and faster interfacial adsorption, whereas higher PC enhances interfacial viscoelasticity, creating a synergistic effect that maximizes both foam expansion and stability. This highlights the importance of tailoring molecular characteristics and composition to optimize the functional performance of SSPS in aerated food systems.

## 4. Conclusions

This systematic study highlights the critical influence of MW and PC on the interfacial adsorption behavior, interfacial rheological properties, and ultimate foam performance of SSPS. A series of SSPS samples with a range of MW and PC was prepared, revealing that low-MW fractions—particularly the LH sample (low MW, high PC)—exhibited the highest foam expansion (FE = 272.5%). This superior performance can be attributed to their smaller particle size, lower surface tension, and reduced ζ-potential, which collectively facilitated rapid adsorption at the air–water interface. While the initial reduction in interfacial tension promotes foam formation, long-term foam stability was found to depend critically on the mechanical strength and viscoelasticity of the interfacial film. Interfacial rheological measurements demonstrated that all SSPS samples formed elastic-dominated films (G′ > G″). Among them, the HM sample (high MW, moderate PC) displayed the fastest increase in G′ and the highest structural strength, suggesting that higher-MW fractions contribute to robust film formation. Within the low-MW group, the LH sample exhibited the strongest elastic response (lowest tan δ) and relatively higher foam stability (FS = 69.9%), benefits attributed to its high PC enhancing interfacial viscoelasticity. Amplitude sweep tests further confirmed that the HM sample possessed the widest linear viscoelastic region, indicating that its interfacial film could withstand larger strains while maintaining structural integrity. Collectively, these results illustrate that foam stability is a multifactorial property, governed not only by the kinetics of interfacial tension reduction but also by the mechanical responsiveness of the interfacial film under stress. This study provides deeper insights into the structure–function relationships of SSPS as a foam stabilizer and offers a theoretical foundation and practical guidance for the rational design of SSPS-based products tailored to specific food systems.

## Figures and Tables

**Table 1:** Extraction parameters and protein contents of nine SSPS samples [14].

Sample	Extraction Conditions				* PC (%)
	Meal/Okara	Temperature (°C)	Time (h)	pH	
High MW-high PC (HH)	1:3	110	2.5	4.0	11.2 ± 0.2
High MW-moderate PC (HM)	1:10	110	2.5	4.0	7.5 ± 0.1
High MW-low PC (HL)	0:1	110	2.5	4.0	3.8 ± 0.2
Moderate MW-high PC (MH)	1:3	120	2.5	4.0	11.5 ± 0.2
Moderate MW-moderate PC (MM)	1:10	120	2.5	4.0	8.5 ± 0.2
Moderate MW-low PC (ML)	0:1	120	2.5	4.0	4.0 ± 0.1
Low MW-high PC (LH)	1:3	130	2.5	4.0	11.5 ± 0.2
Low MW-moderate PC (LM)	1:10	130	2.5	4.0	8.3 ± 0.2
Low MW-low PC (LL)	0:1	130	2.5	4.0	3.9 ± 0.1

* The protein content (PC) was determined by the Kjeldahl method.

**Table 2:** Molecular weight composition of SSPS samples.

Sample	MW (kDa)	Molecular Weight Distribution Range (%)			
		>400 kDa	100~400 kDa	10~100 kDa	<10 kDa
HH	479	32.3	12.9	53.7	1.1
HM	490	41.2	20.2	38.0	0.7
HL	487	40.6	22.1	36.5	0.8
MH	385	22.3	21.1	55.5	1.1
MM	379	25.6	27.4	46.2	0.8
ML	372	23.6	27.7	47.9	0.9
LH	207	15.7	24.3	58.9	1.1
LM	198	16.7	28.1	54.1	1.1
LL	203	15.6	29.8	53.4	1.2

**Table 3:** Amino acid composition of SSPS samples.

Amino Acid (%)	HH	HM	HL	MH	MM	ML	LH	LM	LL
Asp	14.67	13.79	12.98	12.98	13.06	13.00	13.09	14.77	16.64
Glu	22.61	25.63	25.64	22.17	25.56	28.07	23.02	27.42	31.65
Ser	4.82	4.42	4.17	4.54	4.12	4.07	4.66	4.28	2.63
His	4.51	4.09	3.56	3.91	3.70	3.39	4.40	3.45	2.46
Gly	4.91	4.46	4.51	4.72	4.35	4.31	4.88	4.47	4.99
Thr	5.05	4.65	4.59	4.56	4.01	3.71	4.59	3.83	3.24
Arg	5.79	7.00	7.53	6.77	7.92	8.10	6.80	7.25	7.39
* Ala	4.99	4.62	4.74	5.22	4.24	3.84	4.77	3.95	4.34
Tyr	2.14	2.09	1.97	2.43	2.14	1.93	2.08	1.95	2.32
Cys-s	0.39	0.29	0.27	0.17	0.22	0.14	0.16	0.17	0.15
* Val	5.22	4.17	4.19	5.34	4.60	4.09	5.01	4.09	3.81
* Met	0.72	0.48	0.45	0.61	0.45	0.40	0.61	0.46	0.83
* Phe	3.85	3.12	3.18	4.05	4.08	3.73	3.90	3.62	3.44
* Ile	3.54	3.22	3.30	3.76	3.77	3.41	3.60	3.34	2.20
* Leu	5.39	4.63	4.74	6.01	5.65	5.07	5.43	4.86	4.99
Lys	6.86	8.07	8.35	7.55	7.19	7.27	7.46	6.87	4.31
* Pro	4.58	5.32	5.83	5.23	4.95	5.28	5.62	5.21	4.60
hydrophobic amino acids (%)	28.29	25.56	26.43	30.22	27.74	25.82	28.94	25.53	24.21
Total amino acid content (g/100 g)	12.39	8.91	3.15	13.64	10.88	4.38	15.84	10.46	4.63

*: denotes hydrophobic amino acids. Abbreviation: Asp, aspartic acid; Glu, glutamic acid; Ser, serine; His, histidine; Gly, glycine; Thr, threonine; Arg, arginine; Ala, alanine; Tyr, tyrosine; Cys, cysteine; Val, valine; Met, methionine; Phe, phenylalanine; Ile, isoleucine; Leu, leucine; Lys, lysine; Pro, proline.

## Data Availability

The original contributions presented in the study are included in the article, further inquiries can be directed to the corresponding author.

## Abbreviations

The following abbreviations are used in this manuscript:

SSPS	soluble soybean polysaccharide
MW	molecular weight
PC	protein content
FE	foam expansion
FS	foam stability
